# FMRI Study of Neural Responses to Implicit Infant Emotion in Anorexia Nervosa

**DOI:** 10.3389/fpsyg.2017.00780

**Published:** 2017-05-17

**Authors:** Jenni Leppanen, Valentina Cardi, Yannis Paloyelis, Andy Simmons, Kate Tchanturia, Janet Treasure

**Affiliations:** ^1^Department of Psychological Medicine, Institute of Psychiatry, Psychology & Neuroscience, King’s College LondonLondon, UK; ^2^Department of Neuroimaging, Institute of Psychiatry, Psychology & Neuroscience, King’s College LondonLondon, UK; ^3^Department of Psychology, Ilia State UniversityTbilisi, Georgia

**Keywords:** eating disorders, anorexia nervosa, fMRI, emotional infant faces, implicit

## Abstract

Difficulties in social–emotional processing have been proposed to play an important role in the development and maintenance of anorexia nervosa (AN). Few studies, thus far, have investigated neural processes that underlie these difficulties, including processing emotional facial expressions. However, the majority of these studies have investigated neural responses to adult emotional display, which may be confounded by elevated sensitivity to social rank and threat in AN. Therefore, the aim of this study was to investigate the neural processes underlying implicit processing of positively and negatively valenced infant emotional display in AN. Twenty-one adult women with AN and twenty-six healthy comparison (HC) women were presented with images of positively valenced, negatively valenced, and neutral infant faces during a fMRI scan. Significant differences between the groups in positive > neutral and negative > neutral contrasts were investigated in *a priori* regions of interest, including the bilateral amygdala, insula, and lateral prefrontal cortex (PFC). The findings revealed that the AN participants showed relatively increased recruitment while the HC participants showed relatively reduced recruitment of the bilateral amygdala and the right dorsolateral PFC in the positive > neutral contrast. In the negative > neutral contrast, the AN group showed relatively increased recruitment of the left posterior insula while the HC groups showed relatively reduced recruitment of this region. These findings suggest that people with AN may engage in implicit prefrontal down-regulation of elevated limbic reactivity to positively social–emotional stimuli.

## Introduction

Recent large scale meta-analyses and systematic reviews have found that people with anorexia nervosa (AN) have difficulties in various aspects of social–emotional processing, including theory of mind and accurate interpretation of emotions (Caglar-Nazali et al., 2014; Bora and Köse, 2016). Another recent meta-analysis also found that people with AN show a behavioral pattern of reduced expression of emotions when viewing both positive and negative emotionally provoking stimuli (Davies et al., 2016). Additionally, behavioral studies have reported that people with AN also show greater attentional bias toward threatening emotional facial expressions, such as rejecting and angry faces, and attentional avoidance of positive facial expressions, such as accepting and happy faces (Harrison et al., 2010; Cardi et al., 2013, 2015). Furthermore, people with AN show elevated sensitivity to social rank, and report more submissive behavior and feelings of shame (Troop and Baker, 2008; Cardi et al., 2014b; Troop, 2016). These difficulties have been proposed to play an important role in the development and maintenance of disordered eating in AN, increasing isolation and negative mood (Treasure and Schmidt, 2013). Further investigation of the underlying mechanisms is of interest.

Few studies to date have investigated the neural mechanisms that underlie these difficulties in social–emotional processing in AN. A recent systematic review of the literature found that relative to healthy comparison (HC) participants, people with AN show atypical, reduced recruitment in regions such as lateral and medial prefrontal cortex (PFC) in response to social–emotional behavior (McAdams and Smith, 2015). Additionally, a prospective study by Schulte-Rüther et al. (2012) found that reduced recruitment of the prefrontal regions in response to social–emotional behavior at admission to hospital was associated with poorer outcome. Interestingly, other studies have found that when asked to select negatively valenced social words or view provoking stimuli, such as food images, people with AN show greater recruitment of cortical regions, including the dorsolateral PFC and insula, relative to healthy control (HC) participants (Brooks et al., 2011, 2012a; Miyake et al., 2012; Brooks, 2016). Additionally, a recent study by Fonville et al. (2014) investigated neural response to positive facial expressions in acute AN and found a linear increase in neural activation in the right fusiform gyrus, which was greater in the AN group than the HC group. Taken together, these findings suggest that atypical recruitment of lateral prefrontal, insular, and visual attention regions may underlie difficulties in social–emotional processing in AN.

The above mentioned studies have largely focused on investigating atypical emotional processing in the context of peer, adult social–emotional display, which may be confounded by elevated sensitivity to social rank and threat in AN. It would be of interest to explore whether atypical emotional processing extends across lifespan to, for example pre-language infant emotional display. Adults in general are uniquely attuned to social–emotional signaling from infant faces due to absence of language (Brosch et al., 2007; Parsons et al., 2011; Thompson-Booth et al., 2014). Additionally, relative to adult faces infant faces have been found to be perceived as less threatening, more helpless, and evoke caregiving responses in adults (Berry and McArthur, 1985, 1986; Parsons et al., 2011; Senese et al., 2013). Neuroimaging studies have found that relative to adult faces infant faces strongly activate regions involved in social–emotional processing including the lateral PFC and insula, and regions involved in visual attention, such the fusiform gyrus (Caria et al., 2012; Rocchetti et al., 2014). Furthermore, emotional infant faces have been found to strongly recruit limbic regions including the amygdala, as well as multiple areas in the frontal cortex, including the lateral PFC, in healthy adults (Baeken et al., 2009; Montoya et al., 2012).

A study by Cardi et al. (2014a) found that people with eating disorders (EDs) show anomalies in processing infant emotional display. Interestingly, unlike with emotional adult faces, Cardi et al. (2014a) found no significant differences between the people with EDs and HC participants in attentional bias toward positive and negatively valenced emotional infant faces (Cardi et al., 2015). However, relative to HC participants, people with EDs interpreted the positively valenced infant stimuli to be less positive and reported more subjective negative affect in response to negative infant display (Cardi et al., 2014a). Additionally, the participants with EDs displayed fewer positive facial expressions while viewing a positively valenced infant display (Cardi et al., 2014a). These findings suggest that people with EDs may have a tendency to interpret emotional stimuli in a negative way and display reduced facial affect, which are not restricted to peer, other-adult emotional displays, but extends to less threatening and motivationally salient infant emotional expression. However, to our knowledge no studies to date have investigated the neural responses to infant emotional expression in EDs, which could shed light on the mechanisms that may underlie these atypical responses.

Few studies in mood and anxiety disorders, common co-morbid disorders in AN, have investigated neural responses to motivationally salient emotional infant stimuli (Baeken et al., 2010; Schechter et al., 2012; Wonch et al., 2016). A study investigating neural responses to emotional infant stimuli among people with melancholic depression found increased activation in regions including the ventrolateral PFC (VLPFC) and inferior occipital cortex, while viewing positively valenced infant faces relative to scrambled stimuli (Baeken et al., 2010). Another study found that relative to healthy mothers, mothers with postnatal depression showed greater amygdala response to unfamiliar positively valenced infant faces (Wonch et al., 2016). The authors also found that mothers with postnatal depression showed reduced functional connectivity between the amygdala and insula while viewing positively valenced infant stimuli (Wonch et al., 2016). Additionally, previous work has found that mothers with interpersonal trauma related post-traumatic stress disorder (PTSD) showed increased recruitment of regions involved in emotion processing and regulation, including the posterior insula, amygdala, and dorsolateral PFC (DLPFC), while viewing distressed unfamiliar infants relative to content infants (Schechter et al., 2012). Taken together, these findings suggest that there may be deficits in elevated insular and limbic reactivity, and prefrontal down-regulation in response to motivationally salient infant emotion in these disorders.

The aim of the current study was to investigate the neural correlates that underlie implicit processing of infant emotion in people with AN relative to HC participants. Based on the previous findings from studies investigating neural response to social–emotional stimuli in people with AN outlined above, we hypothesized that we would find atypical increased recruitment of regions involved in emotion down-regulation, namely the bilateral lateral PFC, in response to emotional infant faces. Additionally, based on previous neuroimaging work among people with anxiety and mood disorders we hypothesized that the AN group would show atypical elevated neural response to emotional infant faces in regions associated with emotional processing, including the bilateral amygdala and insula. These hypotheses were investigated with regions of interest approach.

## Materials and Methods

### Participants

Forty-seven adult women took part in the study. Twenty-one women had a current DSM-5 diagnosis of AN. Fifteen women with AN were recruited through advertisements placed on EDs charities websites (BEAT and Succeed) and six women with AN were recruited from the South London and Maudsley NHS Foundation Trust inpatient unit. All AN participants recruited from the inpatient unit were receiving treatment at the time of the study. Twenty-six HCs with a BMI between 18.5 and 25 and no history of psychiatric disorders were recruited amongst King’s College London students and staff who responded to advertisements placed on the university’s website. The Structured Clinical Interview for DSM-5 was used to confirm AN diagnosis, and to screen for psychiatric disorders in the HC group (First et al., 2015). Both groups were matched for age and level of education. Participants were excluded from the study if they were left handed or reported a history of head trauma, neurological disease, uncorrected hearing or visual impairment, acute suicidality, history of or current alcohol or drug abuse, or MRI incompatibility (i.e., implanted medical devices of any kind, history of accidents involving metal, any metal in or on the body that cannot be removed, claustrophobia, pregnancy). Additional exclusion criteria for the AN group included psychotropic medication other than selective serotonin reuptake inhibitors (SSRIs). Prior to taking part in the study, all participants were asked to give written informed consent, and were compensated for their time. The study was conducted in accordance with the latest version of the Declaration of Helsinki (1975, as revised in 2008) and was approved by a local National Research Ethics Service (NRES) committee (11/LO/0373).

### Clinical and Questionnaire Measures

The Eating Disorder Examination Questionnaire (EDEQ), a 36-item self-report measure, was used to assess ED behaviors and attitudes over the past 28 days (Fairburn and Beglin, 1994). In the current study, internal consistency of EDEQ was high, with Cronbach’s alpha of 0.90.

The Depression, Anxiety, and Stress scale (DASS) is a 21-item self-report measure assessing severity of depression, anxiety, and stress over the past week (Lovibond and Lovibond, 1995). In the current study, internal consistency of the DASS was high, with Cronbach’s alpha of 0.97.

### Functional Magnetic Resonance Imaging (fMRI) Procedure

Participants were presented with black and white photographs of prototypical positively valenced (smiling), prototypical negatively valenced (crying), and neutral infant faces during a 12-min fMRI scanning session. The images were matched for physical properties, including size, contrast, and luminosity. The stimuli were acquired from a validated set of infant emotional facial expressions and composed of ten different babies (five female, five male) (Kringelbach et al., 2008). The images were used with approval from the authors.

**FIGURE 1:**
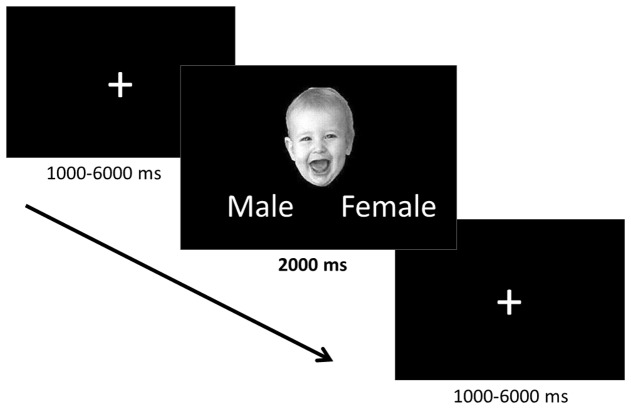
**Implicit emotion processing task.** The positively valenced, negatively valenced, and neutral infant faces were presented one at a time for 2000 ms during which time participants identified the gender of each face. Faces were preceded by a fixation cross the presentation of which was jittered between 1000 and 6000 ms.

The task employed an event-related design, in which the faces were presented one at a time for 2000 ms separated by a fixation cross (**Figure 1**). Ten positively valenced, ten negatively valenced, and ten neutral infant faces were presented in a pseudorandomized order to avoid learning effects and avoid positive and negative faces following one another. The fixation cross inter-stimulus interval was jittered to vary between 1 and 6 s (mean 3.0 s) in order to prevent participants from being able to predict the onset of the trials. While viewing the faces participants were asked to indicate the gender of the faces using the control pad to ensure that they paid attention to the stimuli. The participants were told that the gender identification task would be difficult, to not think about their answers too much, and give their best guess. They were informed that the main goal was to attend to the stimuli and were not given feedback regarding their performance during the task.

### Data Acquisition

The GE Signa 1.5 Tesla scanner (GE Medical Systems, Milwaukee, WI, USA) was used to acquire the magnetic resonance images at the King’s College London, Centre for Neuroimaging Sciences (CNS). An 8-channel radiofrequency birdcage head coil was used to transmit and receive the signal. High resolution T1-weighted magnetization-prepared rapid gradient echo (MP-RAGE) structural images were acquired with a repetition time (TR) of 8.592 ms, and 1.2 mm slice gap, and 1.2 mm slice thickness. One hundred and eighty slices were used to achieve whole brain coverage with an in-plane resolution of 1.25 mm × 1.25 mm. Following the structural scan, a functional blood oxygen level dependent (BOLD) signal was acquired using interleaved T2^∗^ weighted echo planar imaging (EPI) with TR of 2 s, and 0.4 mm slice gap, and 4 mm slice thickness. Thirty slices were used to achieve whole brain coverage with in-plane resolution of 3.75 mm × 3.75 mm. Data quality was assured using an automated quality control procedure (Simmons et al., 1999).

### Statistical Analysis

#### Behavioral and Self-report Data

Behavioral and self-report questionnaire data were analyzed with Stata 14 (StataCorp. 2015, College Station, TX, USA: StataCorp LP.). Group differences in demographic variables and questionnaire responses were investigated using non-parametric median Chi^2^ tests. The behavioral data from the gender identification task was analyzed with 2 × 3 linear mixed models with group (AN, HC) and valence (positive, negative, neutral) entered as fixed effects and a random intercept. Significant interactions were explored with *post hoc* contrasts and pairwise comparisons. *P*-values of 0.05 or lower were considered significant.

#### Functional Neuroimaging Data

FMRI data pre-processing was conducted using SPM8 (Wellcome Department of Cognitive Neurology, Institute of Neurology, London) implemented in MATLAB, version 8.2.0 (Mathworks, Natick, MA, USA). The functional data was corrected for slice-timing and volume-to-volume head motion. The functional data was re-sliced and following re-slicing the voxel size was 1.5 mm × 1.5 mm × 1.5 mm. Following correction the functional images were co-registered to a T1-weighted DARTEL template created from each participant’s high resolution MP-RAGE structural image (Ashburner, 2007). The images were smoothed with an 8 mm FWHM three-dimensional isotropic Gaussian Kernel and normalized to Montreal Neurological institute (MNI) space.

At subject level, the data was modeled using the general linear model framework implemented in SPM8. The BOLD signal was modeled using a single canonical HRF, and the predicted BOLD response was modeled for each of the following conditions: positively valenced faces, negative valenced faces, and neutral faces. Twenty four motion parameters were calculated and used to adjust the time series data for head motion (Friston et al., 1996). Low frequency drift was filtered out of the data using a high-pass filter set to 1/128 s. The subject level model produced the following contrast images: positive faces > neutral faces, and negative faces > neutral faces.

The robust regression toolbox (Wager et al., 2005)^1^ implemented in Matlab 2016b was used to conduct group level random effects analysis. The robust regression toolbox uses iteratively re-weighted least squares (IRLS), which identifies and outweighs influential, extreme outliers. Thus, the IRLS analysis minimizes the impact of extreme outliers and reduces the likelihood of false positive and negative findings with no reduction in power (Wager et al., 2005; Fritsch et al., 2015). We chose this approach to avoid false findings arising from head motion, scanner-related artifacts, or individual participants who were particularly depressed, anxious, stressed, or otherwise vastly differed from the rest of the sample during the MRI scan, which could have led them to process the social–emotional stimuli in a different way (Leppänen et al., 2004; Wager et al., 2005; Kircanski et al., 2014).

The contrast images were first entered into region of interest (ROI) analyses to investigate *a priori* hypothesis within the following bilateral regions: amygdala, insula, and lateral PFC. Anatomical masks of these regions were created using the WFU Pickatlas implemented in SPM8. In the IRLS analysis group was added as a contrast coded covariate (1, -1: AN, HC) with positive test statistics indicating relatively increased activation in the AN group and negative test statistics indicating relatively increased activation in the HC group. The ROI findings were corrected for multiple comparisons with a voxel-wise non-parametric permutation test as recommended by Eklund et al. (2016) (α < 0.05). The permutation test uses the max T distribution to identity a critical threshold to control for family-wise error rate. The permutation test was conducted with 10000 iterations.

Where significant group differences were present, mean signal change data was extracted from the clusters. The data was entered into further analysis to investigate if the atypical activation within the AN group correlated with BMI, duration of illness, medication status, DASS total score, or EDEQ total score. The correlational analyses were conducted using Spearman’s rho in Matlab 2016b.

Whole brain exploratory analysis was conducted to investigate brain responses to positive and negative infant faces in AN. As above, in order to explore differences in brain responses between AN and HC groups, group was added as a contrast coded covariate (1, -1: AN, HC) in the IRLS analyses. The following contrasts were included in the whole brain analysis: positive faces > neutral faces and negative faces > neutral faces. The whole brain analysis was corrected with voxel-wise False Discovery Rate (FDR) thresholded at *q* < 0.05. Effect size, and lower and upper bound 99.9% confidence interval maps for the whole brain analyses were generated using the EScalc toolbox implemented in Matlab^2^ (Gao and Zang, 2015), and are presented in **Supplementary Figure S1** (Positive > Neutral) and **Supplementary Figure S2** (Negative > Neutral).

## Results

### Group Characteristics

Demographic and clinical characteristics of both groups are presented in **Table 1**. Participants did not differ significantly in age or level of education. However, as expected, the AN group had significantly lower BMI and reported higher depression, anxiety, stress, and ED psychopathology than the HC group. Medication status provides the number and the percentage of AN participants taking SSRIs during the time of the study.

**Table 1:** Sample clinical and demographic characteristics.

	AN (*N* = 21) Median [Q1, Q3]	HC (*N* = 26) Median [Q1, Q3]	*X*^2^ (DF) statistic, *p*-value
Age	25.00 [20.00, 34.50]	25.50 [23.00, 28.00]	*X*^2^ (1) = 0.001, *p* = 0.969
Level of education (years)	16.00 [14.00, 17.00]	17.00 [14.00, 20.00]	*X*^2^ (1) = 0.09, *p* = 0.766
BMI	15.84 [14.8, 16.76]	19.90 [19.38, 21.88]	*X*^2^ (1) = 24.68, *p* < 0.001
Medication status *N* (%)	12 (57%)	–	–
EDEQ total	3.87 [3.41, 5.02]	0.31 [0.14, 0.58]	*X*^2^ (1) = 34.04, *p* < 0.001
EDEQ restraint	4.20 [3.60, 5.20]	0.20 [0.00, 0.60]	*X*^2^ (1) = 30.39, *p* < 0.001
EDEQ eating concern	3.90 [3.20, 4.40]	0.00 [0.00, 0.20]	*X*^2^ (1) = 37.38, *p* < 0.001
EDEQ weight concern	3.80 [3.00, 5.50]	0.40 [0.00, 0.60]	*X*^2^ (1) = 27.40, *p* < 0.001
EDEQ Shape concern	4.75 [3.44, 5.69]	0.63 [0.25, 1.13]	*X*^2^ (1) = 37.38, *p* < 0.001
DASS total	67.00 [54.00, 88.00]	9.00 [4.00, 12.00]	*X*^2^ (1) = 31.93, *p* < 0.001
DASS anxiety	19.00 [13.00, 23.00]	0.00 [0.00, 2.00]	*X*^2^ (1) = 25.57, *p* < 0.001
DASS depression	24.00 [18.00, 35.00]	2.00 [0.00, 4.00]	*X*^2^ (1) = 25.57, *p* < 0.001
DASS stress	28.00 [19.00, 34.00]	4.00 [2.00, 10.00]	*X*^2^ (1) = 25.57, *p* < 0.001


*AN, anorexia nervosa; HC, healthy comparison; BMI, body mass index; EDEQ, Eating Disorder Examination Questionnaire; DASS, Depression, Anxiety, and Stress Scale; Medication status, percentage of AN participants taking anti-depressants; Q1, first quartile, Q3, third quartile, DF, degrees of freedom.*

### Task Performance

The gender identification task performance in the AN and HC groups is presented in **Table 2**. Regarding accuracy, the mixed effects model revealed a significant effect of trial with participants performing better in the neutral trials than positive trials (*Z* = 24.97, *p* < 0.001, 95% CI [0.43, 0.50]) or negative trials (*Z* = 24.32, *p* < 0.001, 95% CI [0.44, 0.52]). There were no significant differences between participants performance on the positive and negative trials (*Z* = -0.95, *p* = 0.340, 95% CI [-0.06, 0.02]).

**Table 2:** Gender identification task performance in the AN and HC groups.

	Trial	AN (*N* = 21)	HC (*N* = 26)	*X*^2^ (DF) statistic, *p-*value
Accuracy (%)	Positive	27.50 (9.80)	30.19 (8.42)	Group: *X*^2^ (1) = 0.13, *p* = 0.717
	Negative	28.00 (7.68)	27.50 (7.78)	Trial: *X*^2^ (2) = 832.59, *p* < 0.001
	Neutral	73.55 (7.61)	72.73 (5.29)	Group × Trial: *X*^2^ (2) = 0.85, *p* = 0.653
RT (ms)	Positive	1197.72 (433.12)	1094.15 (182.48)	Group: *X*^2^ (1) = 13.68, *p* = 0.0002
	Negative	1254.36 (352.47)	1172.30 (190.39)	Trial: *X*^2^ (2) = 13.29, *p* = 0.001
	Neutral	1194.57 (392.48)	1070.32 (154.87)	Group × Trial: *X*^2^ (2) = 0.94, *p* = 0.624


*AN, anorexia nervosa; HC, healthy comparison; RT, reaction time, DF, degrees of freedom.*

Similarly, the mixed effects model also revealed a significant effect of trial in participants’ reaction times (**Table 2**). Participants were significantly faster in the neutral trials than positive trials (*Z* = -3.59, *p* < 0.001, 95% CI [-125.03, -36.75]) and positive trials than negative trials (*Z* = -2.62, *p* = 0.009, 95% CI [-117.78, -17.01]). There were no significant differences between participants reaction times in the neutral and negative trials (*Z* = -0.61, *p* = 0.540, 95% CI [-56.68, 29.69]). There was also a significant effect of group in participants’ reaction times (**Table 2**), with HC participants responding significantly faster than AN participants across trials (*Z* = -3.70, *p* < 0.001, 95% CI [-158.04, -48.55]).

### Regions of Interest Findings

**FIGURE 2:**
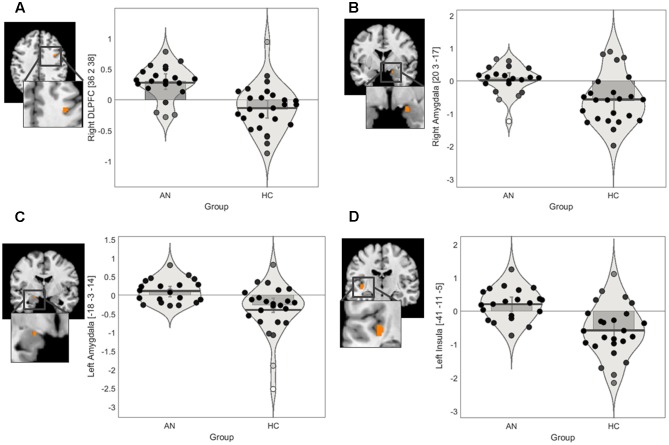
**Regions of interest findings in AN and HC participants.** **(A)** Statistical parametric map of the right lateral PFC ROI with parameter estimate bar plot showing mean activation in the right DLPFC cluster in the positive > neutral contrast. **(B)** Statistical parametric map of the bilateral amygdala ROI with parameter estimate bar plot showing mean activation in the right amygdala cluster in the positive > neutral contrast **(C)** Statistical parametric map of the bilateral amygdala ROI with parameter estimate bar plot showing mean activation in the left amygdala cluster in the positive > neutral contrast. **(D)** Statistical parametric map of the bilateral insula ROI with parameter estimate bar plot showing mean activation in the left posterior insula cluster in the negative > neutral contrast. The *Y*-axis shows mean contrast signal change.

We conducted ROI IRLS analyses to investigate group differences in activation in response to positively valenced and negatively valenced infant faces within the following masks: amygdala, insula, and lateral PFC. The ROI findings for group differences are presented in **Figure 2** and **Table 3**.

**Table 3:** Regions of interest findings in AN and HC participants.

	Peak MNI coordinates			Contrast signal change				
					
Contrast	*x*	*y*	*z*	AN *Mean* (*SE*)	HC *Mean* (*SE*)	Max T statistic, *p*-value	*k*	ROI
Positive > Neutral	-18	-3	-14	0.10 (0.07)	-0.27 (0.10)	*t* = 3.85, *p* = 0.0008	24	Amygdala
	20	3	-17	0.09 (0.08)	-0.60 (0.15)	*t* = 3.54, *p* = 0.0002	14	Amygdala
	36	2	38	0.30 (0.06)	-0.15 (0.07)	*t* = 4.17, *p* < 0.0001	4	DLPFC
Negative > Neutral	-41	-11	-5	0.20 (0.11)	-0.58 (0.17)	*t* = 4.18, *p* = 0.0001	24	Insula


*AN, anorexia nervosa; HC, healthy comparison; ROI, region of interest; MNI, Montreal Neurological Institute; DLPFC, dorsolateral prefrontal cortex; SE, standard error of mean; k, number of voxels; voxel size 1.5 mm × 1.5 mm × 1.5 mm.*

#### Positive > Neutral

The ROI findings revealed a significant difference between the groups in recruitment of a very small cluster in the right DLPFC in the positive > neutral contrast (**Table 3**). Exploration of the mean contrast signal change suggested that the group differences in the DLPFC was driven by relatively increase in recruitment in the AN group in this contrast (**Figure 2A**). Similarly, a significant difference between the groups in recruitment of the bilateral amygdala in the positive > neutral contrast was observed (**Table 3**). Exploration of the mean contrast signal change suggested that the group difference observed in the amygdala was driven by relatively increased recruitment in the AN group and reduced recruitment in the HC group in this contrast (**Figures 2B,C**).

*Post hoc* correlational analysis within the AN group revealed that the mean contrast signal change in the DLPFC and amygdala ROIs did not significantly correlate with psychopathology, medication status, BMI, or duration of illness in this contrast (Supplementary Table S1).

#### Negative > Neutral

There was a significant difference between the AN and HC groups in recruitment of the left posterior insula in the negative > neutral contrast (**Table 3**). Exploration of the mean contrast signal change revealed that the group difference was brought on by relatively increased recruitment in the AN group and reduced recruitment in the HC group in this contrast (**Figure 2D**).

*Post hoc* correlational analysis within the AN group revealed that the mean contrast signal change in the insula ROI did not significantly correlate with psychopathology, medication status, BMI, or duration of illness in this contrast (Supplementary Table S1).

### Whole Brain Findings

The positively valenced faces > neutral faces and negatively valenced faces > neutral faces contrasts did not yield areas of significant group differences in the whole brain exploratory search.

## Discussion

The aim of the current study was to investigate differences in neural mechanisms that underlie implicit processing of emotional infant faces between AN and HC participants. As hypothesized, group differences emerged in the recruitment of the left posterior insula, bilateral amygdala, and the right DLPFC between the AN and HC groups. A very small cluster in the right DLPFC showed increased recruitment in the AN group, but not the HC group, while viewing positively valenced infant faces relative to neutral faces. Interestingly, the bilateral amygdala showed relatively reduced recruitment in the HC group, but not the AN group, in this contrast. While viewing negatively valenced faces relative to neutral faces, the AN groups showed increased recruitment of the left posterior insula, while the HC group showed relatively reduced recruitment of this region. The whole brain analysis did not reveal any further regions showing significant differences between the groups in either contrast.

The present ROI findings revealed increased recruitment of a very small cluster in the right DLPFC in the AN group relative to the HC group when viewing images of positively valenced infant faces. Similar increased recruitment of the lateral PFC has also been found in women with melancholic depression while viewing positively valenced infant faces (Baeken et al., 2010). Increased recruitment of the right DLPFC has also been found among people with depression while viewing salient, positive emotional stimuli, including smiling faces (Jaworska et al., 2015). Further, one study reported a positive correlation between recruitment of the right DLPFC and valence rating of emotional stimuli among people with depression, whereas an inverse correlation was observed among healthy individuals (Grimm et al., 2008). Indeed, increased recruitment of the right DLPFC is often reported among healthy participants during working memory tasks, and while processing negative emotional stimuli particularly when explicitly asked to down-regulate subjective negative emotional responses to intense distressing stimuli (Goldin et al., 2008; Wager et al., 2008; Ochsner et al., 2012; Criaud and Boulinguez, 2013). Similarly, increased lateral and medial prefrontal activation has been reported among healthy women in response to negatively valenced infant stimuli, including images of distressed infants and the sound of infant cry (Baeken et al., 2009; Montoya et al., 2012). Thus it appears that in the healthy population the DLPFC activation is as associated with a form of top-down control to regulate emotional responses to negative stimuli. However, it seems that in AN as well as depression this mechanism may be activated in response to positive emotional stimuli. Still, it is of importance to note that the cluster in the right DLPFC in the present study was very small and replication of these findings is necessary before firm conclusions can be drawn.

The present study also found relatively reduced recruitment of the bilateral amygdala among HC participants, but not among AN participants, while viewing positively valenced infant faces. Reduced recruitment of the bilateral amygdala has also previously been found among healthy individuals while processing positive emotional adult faces (Cowdrey et al., 2012). Interestingly, similar reduced recruitment of the amygdala in response to positive facial expressions has also been reported in people who have recovered from AN (Cowdrey et al., 2012). Additionally, relatively increased recruitment of the amygdala has been found previously among mothers with postnatal depression while viewing positive infant stimuli (Wonch et al., 2016). Furthermore, a number of studies have found that in addition to increased recruitment of the right DLPFC, people with depression show relatively increased recruitment of the amygdala while processing salient, positive emotional stimuli (Jaworska et al., 2015). One study also reported correlated increase in the recruitment of the bilateral amygdala and right DLPFC in people with depression while processing positive emotional facial expressions (Liao et al., 2012). Moreover, as with people recovered from AN, a few studies investigating differences in neural activation in response to positive social–emotional stimuli between people recovered from depression and HC participants have reported no significant differences in the recruitment of the amygdala (Dutra, 2012; Kerestes et al., 2012). Taken together these findings suggest that atypical activation of the bilateral amygdala while processing salient positive social–emotional stimuli may be related to acute state of illness in AN and depression.

A possible interpretation of the above findings is that people with acute AN may engage in implicit prefrontal down-regulation of elevated amygdala reactivity to positively valenced infant stimuli. Indeed, the wealth of previous work in depression has suggested that increased recruitment of the DLPFC and the amygdala in response to positive emotional stimuli to be linked with attempts to down-regulate positive mood (Kupfer et al., 2012). Previous studies have also found that people with AN show greater implicit cognitive control relative to HC during working memory tasks demonstrated by equivalent behavioral performance, but elevated DLPFC activation (Brooks et al., 2012b; Brooks, 2016). This interpretation would be in line with findings from previous behavioral studies reporting reduced facial expressivity in people with AN while viewing positive infant stimuli (Cardi et al., 2014a). Furthermore, these findings are also supported by the steady accumulation of studies reporting reduced facial expressivity in response to general emotionally provoking positive films among people with AN (Davies et al., 2016). Positive facial expressivity has also been found to correlate positively with BMI and negatively with ED psychopathology among people with acute AN (Dapelo et al., 2016; Lang et al., 2016). People recovered from AN, on the other hand, do not display similar reduced facial expressivity to emotionally provoking positive stimuli (Davies et al., 2013). However, as the cluster in the right DLPFC in the present study was very small, replication of these findings with a larger sample is necessary before firm conclusions can be drawn.

The present findings also revealed relatively increased recruitment of the left posterior insula in the AN group and relatively reduced recruitment of this region in the HC group when viewing negatively valenced infant faces. Similar pattern of increased recruitment of the left posterior insula has been seen in mothers with interpersonal trauma related PTSD while viewing videos of distressed unfamiliar infants (Schechter et al., 2012). The increased recruitment of the posterior insula was also associated with increased levels of subjective distress as reported by these mothers, which has been speculated to indicate emotion dysregulation and difficulties in down-regulating subjective negative emotions such as feelings of distress and helplessness (Schechter et al., 2012). Furthermore, increased recruitment of posterior insula has been previously found in response to intense sadness and distress in healthy and depressed individuals, with recovery from depression being marked by decreased recruitment of the posterior insula (Mayberg et al., 1999).

Thus, a possible interpretation of this finding is that people with AN may experience greater subjective distress while viewing salient, negative infant stimuli. Indeed, the posterior insula has been suggested to play an important role in processing of emotional salience and interoceptive awareness (Craig, 2009; Menon and Uddin, 2010; Duerden et al., 2013). Hyperactivation of this region in response to negative emotional stimuli has been associated with subjective feelings of distress among healthy and clinical populations (Mayberg et al., 1999; Schechter et al., 2012). This would be in line with behavioral studies showing that people with AN report generally elevated social anxiety and distress, and report more subjective negative affect in response to emotionally provoking negative stimuli (Gilboa-Schechtman et al., 2006; Lang et al., 2016). Furthermore, one study found that subjective distress significantly mediated difficulties in emotional awareness and attention toward emotions in AN (Gilboa-Schechtman et al., 2006). Taken together, these findings suggest that posterior insula may play an important role in processing negative emotional information in AN and further exploration of this regions in context of subjective negative affect would be of interest.

### Clinical Implications

The current findings are in line with previous behavioral studies suggesting that people with AN may engage in implicit down-regulation of emotions and report elevated subjective distress while viewing particularly salient positive and negative stimuli, respectively (Cardi et al., 2014a; Davies et al., 2016). Suppression of emotional responses can have generally negative emotional and social consequences, including elevated negative mood and social isolation (Gross, 2002; Szczurek et al., 2012). Furthermore, reduced emotional expression in response to infant emotional display can have profoundly disruptive effect on the infant as demonstrated with the still face paradigm (Weinberg and Tronick, 1994) and this may ultimately impact the development of the children of mothers with AN (Micali et al., 2014). Thus, these findings further highlight the need for interventions that target atypical social–emotional processing, including down-regulation of positive emotions and elevated subjective distress, in AN. Pending replication, the present findings may serve as useful targets to assess effectiveness of such interventions.

### Limitations

The main limitation of this study was the gender identification task chosen to ensure that participants paid attention to the stimuli and allow exploration of neural mechanisms underlying implicit emotion processing. Although this task has been previously used successfully with images of adult faces (Fonville et al., 2014), in the present study both groups exhibited poor accuracy in identifying the infants’ gender in the emotional context. Tasks that the participants find too difficult can lead to increased recruitment of other regions in order to try and cope with the task demands or, in some cases, participants may “give up” leading to less time spent on task (Bookheimer, 2000; Huddleston and DeYoe, 2008; Pressman and Gitelman, 2012). Additionally, increasing task demands can influence the processing of social–emotional stimuli leading to reduced recruitment of regions typically involved in emotion processing, such as the amygdala (Blair et al., 2007). Although, in the current study both groups performed equally poorly and they were not given feedback regarding their performance, it is not possible to ascertain that the group differences observed were not partly due to the AN participants feeling guilty about their poor performance. Before firm conclusions can be drawn from the present findings, these results must be replicated with an alternative task and future research should use alternative tasks to further investigate implicit emotion processing.

Although the aim of the present study was to investigate the neutral mechanisms that underlie implicit processing of infant emotional display, it would have been of interest to also investigate neural processes that underlie explicit recognition of emotions in infant faces. There is behavioral evidence that people with EDs show negative bias when interpreting infant emotional displays (Cardi et al., 2014a). Therefore, future studies may benefit from further exploration of the neural processes that underlie such interpretation bias.

Although, emotional infant stimuli have been found to be effective in eliciting strong emotional responses in adults, another limitation of the current study was that we did not evaluate participants’ interest in or experiences with infants, which could influence responses to emotional infant stimuli. For example it has been found that people who are interested in infants display attentional bias toward infant faces over adult faces and are more motivated to view images of infants than those who are not interested in infants (Cárdenas et al., 2013; Charles et al., 2013). Future studies may benefit from assessing participants interest in and experiences with infants with self-report measures, such as the Interest in Infants Inventory (Goldberg et al., 1982; Maestripieri and Pelka, 2002).

Another limitation of the study was small sample size and lack of IQ or cognitive assessment. The small sample size prevented us from exploring further differences between AN participants who were taking SSRIs during the time of the study and those AN participants who were free of psychotropic medication. Additionally, a small number of the AN participants were receiving inpatient treatment during the study, whereas the majority of the group were volunteers and not in treatment for their ED. Therefore, the impact of SSRI medication and treatment on the group differences observed cannot be ruled out. Similarly, we did not conduct formal IQ or cognitive assessment, which could have impacted the findings. However, the AN and HC groups were matched for level of education and the task did not have a strong cognitive component. Still future studies may benefit from including larger samples and assessing the impact of the above-mentioned factors.

Finally, the AN participants in the current study were not weight recovered and therefore, it cannot be ruled out that the group differences were due to state of malnutrition. Malnutrition can, thus pose difficulties in trying to explore the neural mechanisms that underlie social–emotional processing in AN. On the other hand, including only weight recovered AN participants is not without difficulties. Different stages of illness and recovery are often associated with different challenges (Kordy et al., 2002; Treasure et al., 2015), making it difficult to generalize findings from studies including only weight recovered AN participants to people in the acute state of illness. Thus future studies may benefit from exploring the neural processes that underlie difficulties in social–emotional functioning across different stages of illness.

## Conclusion

The aim of the current study was to investigate differences in neural mechanisms that underlie implicit processing of emotional infant faces between AN and HC participants. The results revealed increased recruitment of the bilateral amygdala and a very small cluster in the right DLPFC in the AN group relative to the HC group while viewing positively valenced infant stimuli. Additionally, relative to the HC group, the AN participants showed increased recruitment of the left posterior insula while viewing negatively valenced infant stimuli. These findings suggest that people with AN may engage in increased prefrontal down-regulation of elevated limbic response to salient positive social–emotional stimuli, and may experience elevated subjective distress while viewing salient negative social–emotional stimuli. These neural processes may serve as useful targets for future interventions in AN, although replication of these findings with a larger sample size and an alternative task is necessary before firm conclusions can be drawn.

## Author Contributions

JL, YP, and KT made substantial contributions to the acquisition, analysis, or interpretation of data for the work. VC, AS, and JT made substantial contributions to the conception or design of the work. All authors were involved in drafting the work and revising it critically for important intellectual content, gave final approval of the version to be published, and are in agreement to be accountable for all aspects of the work in ensuring that questions related to the accuracy or integrity of any part of the work are appropriately investigated and resolved.

## Conflict of Interest Statement

The authors declare that the research was conducted in the absence of any commercial or financial relationships that could be construed as a potential conflict of interest.

## Footnotes

1 https://github.com/canlab/RobustToolbox

2 http://restfmri.net

## Supplementary Material

**FIGURE S1 |** **(A)** Effect size (Hedges’ g) map of the positively valenced > neutral infant faces contrast. Positive effect sizes indicate larger mean contrast signal change in the AN group relative to the HC group. Negative effect sizes indicate larger mean contrast signal change in the HC group relative to the AN group. **(B)** 99.9% confidence interval lower bound. **(C)** 99.9% confidence interval upper bound. Red to white colors indicate positive figures, blue to green colors indicate negative figures.

**FIGURE S2 |** **(A)** Effect size (Hedges’ g) map of the negatively valenced > neutral infant faces contrast. Positive effect sizes indicate larger mean contrast signal change in the AN group relative to the HC group. Negative effect sizes indicate larger mean contrast signal change in the HC group relative to the AN group. **(B)** 99.9% confidence interval lower bound. **(C)** 99.9% confidence interval upper bound. Red to white colors indicate positive figures, blue to green colors indicate negative figures.
