# Supplementary figures and images for: FMRI Study of Neural Responses to Implicit Infant Emotion in Anorexia Nervosa
Source: Front Psychol. 2017 May 17;8:780. doi: 10.3389/fpsyg.2017.00780 (PMC5434152; doi:10.3389/fpsyg.2017.00780)

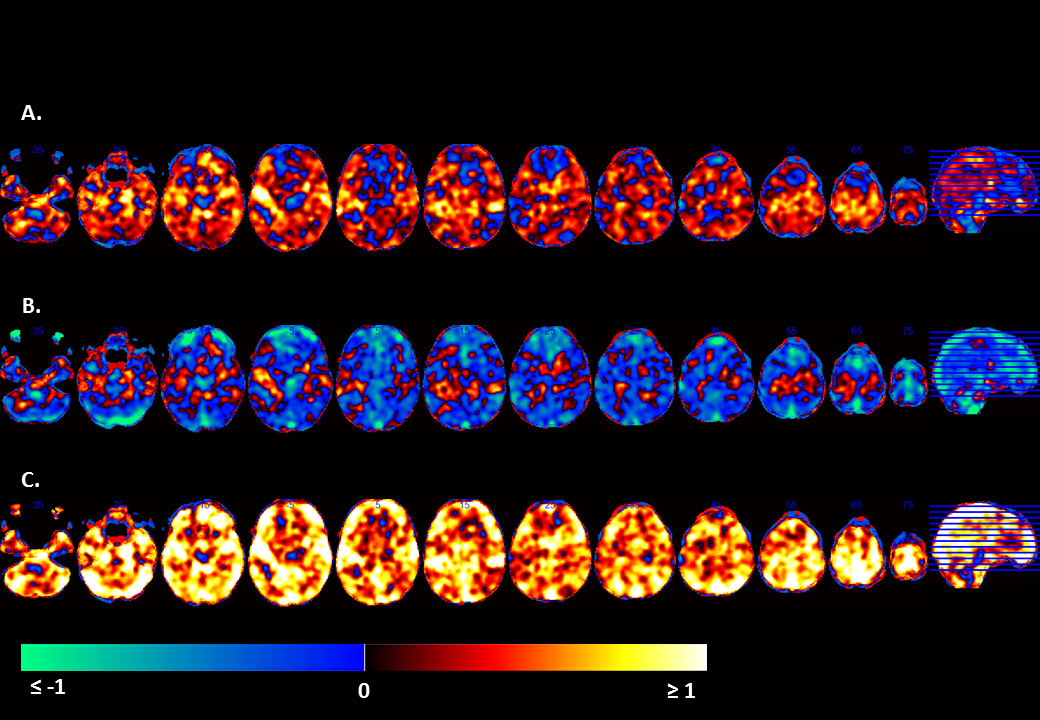

Supplement: Supplementary file 1 [file Image_1.TIF]

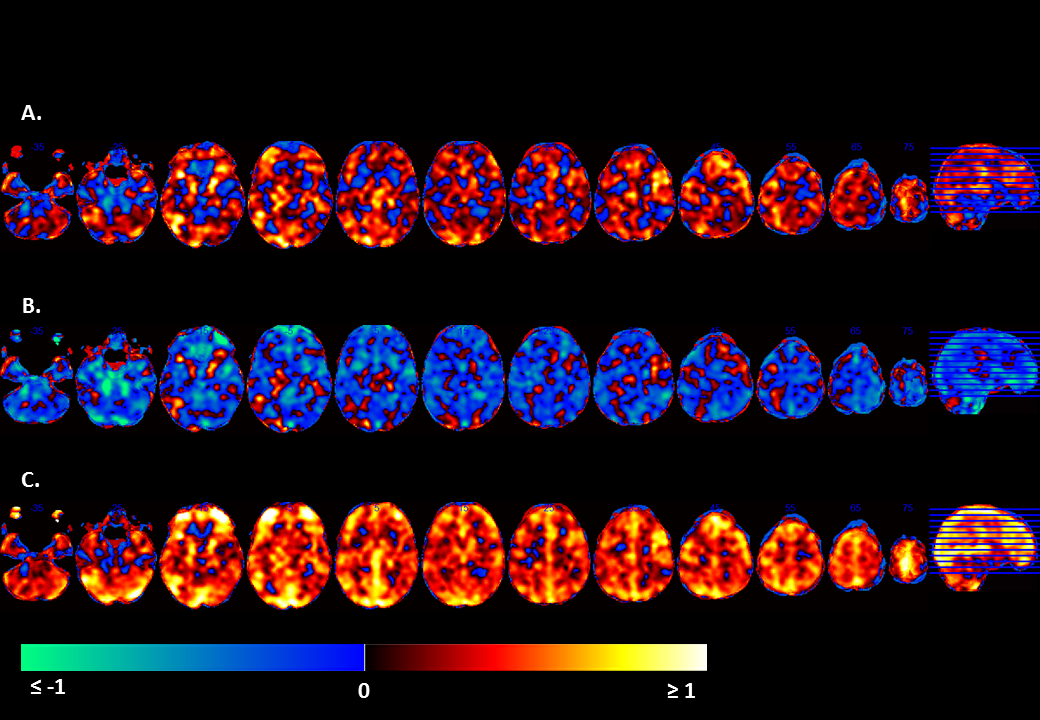

Supplement: Supplementary file 2 [file Image_2.TIF]
