# Supplementary material for: FMRI Study of Neural Responses to Implicit Infant Emotion in Anorexia Nervosa
Source: Front Psychol. 2017 May 17;8:780. doi: 10.3389/fpsyg.2017.00780 (PMC5434152; doi:10.3389/fpsyg.2017.00780)
Supplement: Supplementary file 3 [file Table_1.DOCX]

Supplementary Table 1. Correlations between ROI mean signal change and BMI, medication status, and self-reported psychopathology in the AN group

| Contrast | ROI | BMI | Medication status | EDEQ | DASS |
| --- | --- | --- | --- | --- | --- |
| Positive > Neutral | Left Amygdala | ρ = 0.31, p = 0.188 | ρ = 0.07, p = 0.764 | ρ = 0.30, p = 0.196 | ρ <0.01, p = 0.999 |
|  | Right Amygdala | ρ = 0.13, p = 0.575 | ρ = 0.15, p = 0.519 | ρ = 0.43, p = 0.057 | ρ = -0.14, p = 0.546 |
|  | DLPFC | ρ = -0.17, p = 0.469 | ρ = 0.23, p = 0.337 | ρ = -0.21, p = 0.366 | ρ = -0.08, p = 0.725 |
| Negative > Neutral | Insula | ρ = -0.26, p = 0.259 | ρ = -0.33, p = 0.151 | ρ = -0.24, p = 0.307 | ρ = -0.25, p = 0.292 |

ROI = region of interest; DLPFC = dorsolateral prefrontal cortex; BMI = body mass index; EDEQ = Eating Disorder Examination Questionnaire; DASS = Depression, Anxiety, and Stress Scale
